# Comparative Evaluation of Microwave- and Ultrasound-Assisted Extraction of Antioxidants from Parsley (*Petroselinum crispum* (Mill.) Fuss) and Coriander (*Coriandrum sativum* L.) Processing By-Products

**DOI:** 10.3390/foods15193397

**Published:** 2026-09-23

**Authors:** Joana Solinho, Élia Fernandes, Diana Barros, Manuel Vázquez, Rita Pinheiro

**Affiliations:** 1 School of Technology and Management, Polytechnic Institute of Viana do Castelo, Avenida Atlântico, 4900-348 Viana do Castelo, Portugal; jsolinho@ipvc.pt (J.S.); eliaf@estg.ipvc.pt (É.F.); barrosdiana@estg.ipvc.pt (D.B.); 2 Center for Research and Development in Agrifood Systems and Sustainability (CISAS), Polytechnic Institute of Viana do Castelo, Avenida Atlântico, 4900-348 Viana do Castelo, Portugal; 3 Department of Analytical Chemistry, Faculty of Veterinary, Campus Terra, University of Santiago de Compostela, 27002 Lugo, Spain; manuel.vazquez@usc.es

**Keywords:** food waste valorisation, microwave-assisted extraction, ultrasound-assisted extraction, antioxidant capacity, circular bioeconomy

## Abstract

Aromatic-herb processing generates large volumes of residues that remain rich in bioactive compounds yet are seldom valorised. This study compared the recovery of phenolic compounds and antioxidant capacity from parsley and coriander processing by-products under a fully crossed design combining two extraction methods (microwave-assisted; ultrasound-assisted), two temperatures (60 and 80 °C), two times (15 and 30 min) and three solvents (water, 50% ethanol, and 80% ethanol (*v*/*v*)). For each condition, four separate extractions were performed and combined into a single pooled extract, which was analysed in triplicate; the reported values therefore reflect analytical repeatability, and the between-condition comparisons should be interpreted accordingly. Each of the 24 conditions per species was characterised by total phenolic content (TPC), DPPH, and ABTS. Solvent composition was the dominant factor for both matrices, but in opposite directions of polarity: coriander TPC peaked in water (up to 49.71 ± 0.73 mg GAE/g DW), whereas parsley TPC peaked in 80% ethanol (7.18 ± 0.29 mg GAE/g DW), consistent with differences in the polarity of the extractable constituents. The conditions maximising TPC did not coincide with those maximising radical-scavenging activity, and DPPH and ABTS provided complementary rather than redundant information. In practical terms, coriander residues yielded the highest phenolic content with water, whereas their strongest antioxidant capacity (ABTS) was obtained with 50% ethanol under ultrasound; parsley residues required 80% ethanol with ultrasound. The best-performing condition therefore depended on whether phenolic recovery or antioxidant capacity was the target. These findings demonstrate that botanically related species require distinct, matrix-specific extraction strategies and support the tailored valorisation of aromatic-herb residues within a circular-bioeconomy framework.

## 1. Introduction

The transition towards a circular bioeconomy has placed the valorisation of agri-food by-products at the centre of current food-science research. Food waste is generated at every stage of the supply chain, and processing alone is estimated to account for approximately 19% of total food waste in Europe, with households representing the largest single share [1]. Beyond the environmental burden associated with its disposal, this biomass is chemically valuable: plant-derived residues typically retain substantial quantities of phenolic compounds, carotenoids, tocopherols, and other phytochemicals whose recovery generates value-added products for the food, nutraceutical, and cosmetic sectors while simultaneously reducing waste volumes [2]. The European Union has actively supported this transition through the Green Deal and the Farm to Fork strategy, and a growing number of research programmes are now dedicated specifically to the reduction and valorisation of agri-food residues [1,3].

Aromatic and medicinal plants represent a segment of the horticultural sector in which residue generation is particularly high relative to the marketable fraction. Processing operations such as trimming, sorting, drying, and fresh-cut packaging routinely discard stems, coarse leaves, and off-grade material that are morphologically unsuitable for commercialisation but chemically comparable to the marketed product. Despite this, aromatic-herb residues remain considerably less studied than fruit and vegetable by-products, for which extraction protocols and compositional data are already well established [2,4]. This gap is significant, since the phytochemical richness of aromatic species is precisely what underpins their commercial value.

Parsley (*Petroselinum crispum* (mill.) Fuss) and coriander (*Coriandrum sativum* L.) are two of the most widely cultivated aromatic herbs worldwide, both belonging to the Apiaceae family and both extensively used fresh, dried, or as flavouring ingredients. Their botanical proximity, however, conceals substantial compositional divergence. Parsley is characterised by two distinct chemical domains: a phenylpropanoid-rich essential oil typically dominated by apiol and myristicin, and a phenolic fraction in which the flavone apigenin and its glycoside apiin predominate, accompanied by coumarins and furanocoumarins [5,6,7]. Coriander, in contrast, accumulates a broader spectrum of hydrophilic constituents, including chlorogenic and caffeic acids, quercetin, kaempferol, and rhamnetin derivatives, together with tocopherols, carotenoids, ascorbic acid, and anthocyanins, alongside an essential oil in which linalool and (E)-2-decenal are major components depending on the plant part considered [8,9,10]. These differences in the polarity distribution of the bioactive pool provide a mechanistic basis for expecting the two matrices to respond differently to identical extraction conditions, a hypothesis that has not, to our knowledge, been systematically tested under a common multifactorial design.

The recovery of phenolic compounds from plant matrices depends critically on the extraction technique employed. Conventional solid–liquid approaches such as maceration and Soxhlet extraction are progressively being replaced by so-called green extraction technologies, including microwave-assisted extraction (MAE), ultrasound-assisted extraction (UAE), pressurized liquid extraction, and supercritical fluid extraction, which reduce solvent consumption, processing time, and energy input while improving recovery [10,11]. UAE operates through acoustic cavitation: the propagation of ultrasound waves through the liquid medium generates alternating compression and rarefaction cycles that produce, grow, and collapse microscopic bubbles, releasing intense, localized shear forces together with transient temperature and pressure extremes that mechanically disrupt the plant cell wall and facilitate solvent penetration [11]. MAE, by contrast, relies on the direct interaction of microwave radiation with polar molecules within the matrix, producing rapid volumetric heating, internal pressure build-up, and consequent cell rupture; because of this rapid heating and enhanced mass transfer, MAE typically requires considerably shorter processing times than conventional techniques [12].

The performance of both techniques is nevertheless strongly conditioned by the operating parameters selected, and these parameters do not act independently. Solvent polarity determines which fraction of the phytochemical pool is solubilized: polar solvents such as water and aqueous alcohols preferentially recover hydrophilic phenolic acids and flavonoid glycosides, whereas systems with a higher organic-solvent fraction favour moderately lipophilic constituents including flavonoid aglycones and terpenoid-related compounds [8,13]. Importantly, the relationship between solvent polarity and extraction yield is not linear, and the optimal water–organic ratio has repeatedly been shown to depend on the specific biomass source rather than following a generalizable rule [13,14]. Comparative studies across botanically related species have confirmed that solvent selection significantly affects extraction yield, phenolic content, and antioxidant activity, with the optimum varying between species of the same family [15,16].

Temperature and time exert an equally matrix-dependent influence, acting simultaneously on two opposing processes. Higher temperatures increase solute solubility, reduce solvent viscosity, and accelerate diffusion, thereby enhancing mass transfer; however, they also accelerate the degradation kinetics of thermolabile constituents [12,17]. Phenolic compounds are widely recognised as thermally unstable and prone to loss during processing, and it has been repeatedly demonstrated that a temperature which is not appropriately selected can reduce rather than improve the final yield [17]. In MAE specifically, shorter extraction times limit the degradation of thermally unstable compounds, whereas prolonged exposure may increase gross yield at the expense of compound stability [12]. The practical consequence is that temperature and time must be optimised jointly rather than sequentially, since the optimal duration is itself a function of the temperature applied, an interaction that is frequently reported but seldom explicitly quantified in comparative species studies [18].

A further and often underappreciated consideration concerns the choice of analytical response variable. The Folin–Ciocalteu assay for total phenolic content (TPC), the DPPH radical-scavenging assay, and the ABTS radical cation decolorization assay are among the most widely applied methods for characterising plant extracts, owing to their simplicity, low cost, and modest instrumental requirements [19]. These methods do not, however, measure the same property. TPC quantifies total reducing capacity attributable to phenolic hydroxyl groups, and the molar response of the the Folin–Ciocalteu assay measures the total reducing capacity of the extract rather than phenolics specifically, since the reagent also reacts with other reducing substances present in the matrix; the results are therefore best interpreted as Folin–Ciocalteu-reactive (phenolic and other reducing) compounds [20]. DPPH and ABTS, in turn, both involve hydrogen-atom-transfer and single-electron-transfer mechanisms yet differ fundamentally in the reaction medium they tolerate.

This difference in medium has direct consequences for which compounds each assay detects. The DPPH radical is soluble predominantly in organic solvents, and the assay therefore reflects the contribution of more lipophilic antioxidants; the ABTS radical cation, by contrast, is soluble in both aqueous and organic media, allowing the antioxidant capacity of hydrophilic and lipophilic compounds to be assessed within a single methodological framework. Comparative studies have reported that TPC correlates more strongly with ABTS than with DPPH across a wide range of food matrices, and that the ABTS assay generally provides a more comprehensive estimate of antioxidant capacity in fruits, vegetables, and beverages [21]. Structure–activity analyses have further shown that certain flavonoid subclasses, including some dihydrochalcones and flavanones, react with the ABTS radical but not appreciably with DPPH, and that the response in the DPPH assay is governed largely by the number and positioning of hydroxyl groups, whereas the structure–activity relationship in the ABTS assay is less clearly defined [22]. Consequently, discrepancies between assays are not analytical artefacts to be reconciled but informative signals regarding the polarity distribution and structural composition of the antioxidant pool present in each extract.

For the present study, this methodological consideration is of relevance, given that parsley and coriander are expected to differ precisely in the polarity distribution of their bioactive constituents. Therefore, the parallel application of DPPH and ABTS offers a means of probing that difference directly: a matrix whose antioxidant capacity is dominated by lipophilic constituents should be captured preferentially by DPPH, whereas one dominated by hydrophilic phenolics should be more fully represented by ABTS. Reporting TPC alone, or a single radical-scavenging assay, would therefore risk both underestimating the functional potential of one of the two matrices and obscuring the mechanistic basis of any species-specific extraction optimum identified.

Against this background, the present study was designed to address four interrelated questions. First, how do extraction method (MAE vs. UAE), temperature (60 and 80 °C), time (15 and 30 min), and solvent composition (water, 50% ethanol (*v*/*v*) and 80% ethanol (*v*/*v*)) jointly affect the recovery of phenolic compounds from parsley and coriander processing residues? Second, do these two botanically related species share a common optimal extraction protocol, or do their differing phytochemical profiles impose species-specific optima? Third, do the conditions that maximise total phenolic content coincide with those that maximise radical-scavenging activity within each species? Fourth, do the DPPH and ABTS assays converge in identifying the optimal condition, or do their differing solvent compatibilities reveal complementary information about the composition of the extracted antioxidant pool? By addressing these questions under a single, fully crossed experimental design applied identically to both matrices and evaluated through three complementary assays, this work aimed to provide directly comparable evidence on the matrix-dependence of green extraction protocols and to support the design of tailored valorisation strategies for aromatic-herb processing residues.

## 2. Materials and Methods

### 2.1. Plant Material and Sample Preparation

Parsley (*Petroselinum crispum* (mill.) Fuss) and coriander (*Coriandrum sativum* L.) processing by-products comprising stems, off-grade leaves, and trimmings discarded during commercial fresh-herb preparation were supplied by Aromáticas Vivas^®^, Viana do Castelo, Portugal during May 2026. All samples were obtained from a single production batch; the results are therefore representative of that batch. Upon reception, the samples were carefully washed with potable water to remove soil and debris, followed by immersion in a sanitizing solution, in a sodium hypochlorite solution (150 ppm free chlorine) for 15 min and a final wash with clean water. Drying was performed at 60 °C in a convective oven (VPE-061, Fagor, Mondragón, Spain) until the moisture content was below 10%, which required 8 h for parsley and 4 h for coriander. This temperature was selected on the basis of our own preliminary work on the same two species, in which drying at 60 °C did not reduce the phenolic content or antioxidant capacity relative to a lower drying temperature (40 °C); 60 °C was therefore adopted as a condition that preserves the phenolic content while allowing a substantially shorter process. Dried material was ground in a Termomix (TM31, Vorwerk, Wuppertal, Germany) and subsequently milled and sieved (amplitude of 0.2 for 1 min) using the Analysette 3 PRO machine (Fritsch, Idar-Oberstein, Germany), selecting particles within the diameter range of 200 to 850 µm. The resulting powders were stored in polyamide/polyethylene plastic bags (PA/PE—20/70) (thickness 90 μm; permeability: O_2_—50 cm^3^/m^2^ dbar; CO_2_—150 cm^3^/m^2^ dbar; N_2_—10 cm^3^/m^2^ dbar; water vapor transmission—2.8 g/m^2^ d) and storage at room temperature, protected from light and moisture, until extraction. The by-products consisted of stems, leaves, and trimmings combined in a fixed, consistent proportion across all samples, approximately 50% leaves, 50% stems.

### 2.2. Experimental Design

A multifactorial experimental design was applied independently to each plant matrix (parsley and coriander), combining four extraction variables: (i) extraction method, microwave-assisted extraction (MAE) or ultrasound-assisted extraction (UAE); (ii) extraction temperature, 60 or 80 °C; (iii) extraction time, 15 or 30 min; and (iv) solvent composition, water (W), 50% (*v*/*v*) aqueous ethanol or 80% (*v*/*v*) aqueous ethanol. This resulted in 24 experimental conditions per species (2 methods × 2 temperatures × 2 times × 3 solvents). For each condition, four separate extractions were performed and combined into a single pooled extract, which was then analysed in triplicate for every assay (TPC, DPPH, and ABTS). The reported values represent the mean of three analytical replicates of the pooled extract per condition.

Every extract was subsequently characterised by three complementary assays—TPC, DPPH, and ABTS. The temperature levels were selected to span the range most reported as effective for polyphenol recovery from plant matrices while remaining below the threshold at which extensive thermal degradation is expected [14]. The solvent series was chosen to cover a broad polarity gradient, from purely aqueous to predominantly ethanolic, since the best-performing condition among the tested water–ethanol ratios is known to be biomass-specific [13]. Ethanol was selected as the organic modifier in preference to methanol or acetone on the grounds of food-grade compatibility and its GRAS status, consistent with green extraction principles [4]. The parallel use of DPPH and ABTS was adopted deliberately, given the differing solvent compatibility of the two radicals and the consequent difference in the fraction of the antioxidant pool each can detect.

Samples were coded following the scheme Species_Temperature_Time_Method_Solvent, where S denotes parsley and C denotes coriander. For example, S_60_15_MAE_W designates parsley extracted at 60 °C for 15 min by MAE using water, while C_80_30_UAE_80 designates coriander extracted at 80 °C for 30 min by UAE using 80% ethanol (*v*/*v*).

### 2.3. Microwave-Assisted Extraction (MAE)

MAE was performed using a Microwave Extraction System, (ETHOS X, SK-12 medium pressure rotor, Milestone, Sorisole, Italy), equipped with temperature and pressure control specifications, utilising advanced temperature and pressure monitoring to maintain optimal and safe reaction conditions. For each extraction, 10 g of dried, ground residue was combined with 200 mL of the corresponding solvent. A solid-to-liquid ratio of 1:20 (*w*/*v*) was fixed to isolate the four studied factors and was therefore not included in the optimization design; thus, whether it represents the absolute optimal level for each by-product remains to be established. Nevertheless, this ratio was selected based on previous literature for similar plant matrices, as it provides an adequate concentration gradient for efficient mass transfer of phenolic antioxidants without causing premature solvent saturation or excessive extract dilution, while minimising overall solvent consumption and waste [23].

Vessels were subjected to microwave irradiation at 1600 W with a ramp time of 10 min to the target temperature (60 or 80 °C), which was then held for the defined extraction time (15 or 30 min) under continuous magnetic stirring.

Following extraction, vessels were cooled to room temperature before opening. Extracts were filtered through Whatman No. 1 filter paper/0.90 µm membrane, under vacuum and diluted to 200 mL with the respective extraction solvent. The extracts were stored at 4 °C until analysis, which was performed within 24 h to minimise post-extraction degradation.

### 2.4. Ultrasound-Assisted Extraction (UAE)

UAE was conducted using an ultrasonic bath (SONOREX DIGITEC DT 1028, Scansci, Portugal) with a maximum power of 1200 W, with an ultrasonic frequency of 35 kHz. The solid-to-liquid ratio was identical to that described for MAE (Section 2.3).

Extraction temperature was maintained at 60 or 80 °C, with temperature monitored continuously using a calibrated thermocouple. Sonication was applied for 15 or 30 min according to the experimental design, in continuous mode. Post-extraction handling, filtration, and storage were performed exactly as described in Section 2.3.

The two techniques operated at different nominal powers (MAE, 1600 W; UAE, 1200 W); consequently, the ‘method’ factor combines the extraction mechanism with a difference in applied power, and the method effects should be interpreted as the joint outcome of both rather than as a mechanism-only comparison.

### 2.5. Determination of Total Phenolic Content (TPC)

The total phenolic content of each extract was determined by the Folin–Ciocalteu colorimetric method [20], with minor modifications. As the Folin–Ciocalteu reagent is not specific to phenolic compounds and responds to other reducing species, results are expressed as Folin–Ciocalteu-reactive compounds in gallic acid equivalents. As the Folin–Ciocalteu reagent also reacts with non-phenolic reducing substances, the values are best interpreted as Folin–Ciocalteu-reactive (phenolic and other reducing) compounds rather than as a specific measure of the phenolic pool.

Briefly, 100 µL of extract (or gallic acid standard, or blank) was mixed with 200 µL of Folin–Ciocalteu reagent (0.2 M) and 800 µL of aqueous sodium carbonate solution (Na_2_CO_3_, 7% *w*/*v*). The mixture was homogenised and kept in the dark at room temperature for 2 h. Then, 200 µL of each solution was transferred to a transparent 96-well microplate, and the absorbance was measured at 765 nm using a Varioskan LUX Multimode Microplate Reader (Thermo Scientific, Vantaa, Finland). A calibration curve was prepared with gallic acid in water (0, 5, 10, 20, 40, 60, 80, 100, 120, and 160 mg/L). As the Folin–Ciocalteu reagent is not specific to phenolic compounds and responds to other reducing species, results are expressed as Folin–Ciocalteu-reactive compounds in mg gallic acid equivalents (GAE)/g DW.

### 2.6. DPPH Radical-Scavenging Assay

The antioxidant activity of the extracts was evaluated using the DPPH (2,2-diphenyl-1-picrylhydrazyl) free-radical scavenging assay [24], using Trolox as standard. A methanolic DPPH solution (60 µM) was prepared. For each extract, at least three dilutions were prepared in triplicate (adjusted with methanol). In a transparent 96-well microplate, 50 µL of each dilution was mixed with 200 µL of the DPPH solution, and the plate was kept in the dark for 30 min. Absorbance was measured at 520 nm using methanol as blank, on a Varioskan LUX Multimode Microplate Reader (Thermo Scientific, Vantaa, Finland). A calibration curve was prepared with Trolox in methanol (20, 40, 60, 80, 100, 120, 200, 300, and 400 µM), and results were expressed as mg Trolox equivalents (TE)/g DW.

### 2.7. ABTS Radical Cation Decolorization Assay

Antioxidant capacity was additionally determined by the improved ABTS (2,2′-azino-bis (3-ethylbenzothiazoline-6-sulphonic acid) radical cation decolorization assay [19]. The ABTS radical cation (ABTS•+) was generated by reacting an aqueous ABTS stock solution (7 mM) with potassium persulfate (2.45 mM) and allowing the mixture to stand in the dark at room temperature for 16 h to ensure complete radical formation. Because the radical is pre-formed prior to the addition of the sample, this configuration avoids the interference associated with continuous radical generation in the presence of the antioxidant.

Prior to analysis, the ABTS•+ solution was diluted with methanol to an absorbance of 0.70 ± 0.05 at 735 nm, and the decrease in absorbance at 735 nm was recorded after 6 min of incubation in the dark at room temperature. The results were obtained as mg of Trolox equivalent per g DW.

Because the antioxidant assays were performed in methanolic media whereas the extracts were obtained in water–ethanol systems, solvent exchange may have altered the analyte solubility and thus the measured response; this is a common but recognised limitation, and absolute values across assays with different reaction media should be compared with caution. Despite this limitation, this operational procedure has been widely applied in similar studies on by-product valorisation, as methanol represents the standard reaction medium required to ensure stability and adequate solubilisation [25,26].

### 2.8. Statistical Analysis

Statistical analysis was conducted by TIBCO^®^ Statistica^®^, v.14.0.0, TIBCO Software Inc, Palo Alto, CA, USA. All analytical determinations were performed in triplicate per condition, and results are expressed as mean ± standard deviation of these three analytical replicates. Given that the four extractions per condition were pooled into a single extract before analysis, the following statistical analysis is exploratory and should be interpreted as an indication of trends rather than as confirmatory inference; the identified effects would require independent extraction replicates for formal confirmation. The individual and interactive effects of extraction method, temperature, time, and solvent composition on TPC, DPPH, and ABTS responses were evaluated by ANOVA, with particular attention to the temperature × time and solvent × method interaction terms. Where significant effects were detected, means were compared using Tukey’s HSD post hoc test. For the same reason, effect sizes and correlation coefficients among the assays were not computed, as these would have required independent extraction replicates to be meaningful; the interaction effects are therefore presented and discussed as observed trends rather than quantified magnitudes. Where interactions are described, the corresponding condition means (Table 1 and Table 2) illustrate the direction of these trends.

## 3. Results and Discussion

For coriander, TPC was maximal in purely aqueous extracts, reaching 49.71 ± 0.73 and 49.65 ± 0.92 mg/g under MAE at 60 °C for 15 and 30 min, respectively, with several other water conditions sharing the top statistical group (Table 1). Increasing the ethanol fraction progressively reduced TPC, the lowest values being recorded for the 50% ethanol (*v*/*v*) and 80% ethanol (*v*/*v*) systems (down to 10.14 ± 0.91 mg/g). This behaviour seems to indicate that the phenolic pool of coriander residues is dominated by hydrophilic constituents most efficiently solubilized by water. The finding is fully consistent with the reported phytochemistry of coriander, whose polar extracts are characterised by hydrophilic phenolic acids such as chlorogenic and caffeic acids together with flavonoid glycosides, and with a targeted metabolomic study in which polar solvents yielded both the highest total phenolic content and the highest antioxidant activity [8]. Aqueous coriander extracts have likewise been shown elsewhere to retain substantial phenolic content and appreciable antioxidant capacity [27]. These patterns are consistent with the reported phytochemistry of the two species; however, it must be emphasised that this is an inference based on the response of total phenolics to solvent polarity in the present study and was not confirmed by identification of individual compounds (e.g., by HPLC or LC–MS), which is proposed as future work.

The temperature effect, although statistically significant for coriander TPC (*p* < 0.01), was of small magnitude in the aqueous system and did not alter the ranking of the best conditions, whereas extraction time exerted no significant main effect. This limited role of temperature is in line with reports that, over moderate ranges, the two levels tested may not differ sufficiently to produce large changes in phenolic recovery, and that excessive temperature can even be detrimental through thermal degradation of the more labile phenolics [28]. From a practical standpoint, the near-equivalence of 60 and 80 °C in water favours the lower temperature on energy-efficiency grounds.

Parsley displayed the opposite solvent polarity dependence (Table 2). TPC was highest in the 80% ethanol (*v*/*v*) ethanol system, peaking at 7.18 ± 0.29 and 7.15 ± 0.12 mg/g (80 °C/15 min, MAE and UAE respectively), and was lowest in water, where several conditions fell below 1 mg/g. The overall TPC values were also markedly lower than those of coriander, by approximately sevenfold. The preference of parsley for a more ethanolic medium is consistent with its distinct phytochemistry: parsley is particularly rich in the flavone apigenin and its glycoside apiin, together with a phenylpropanoid-rich essential-oil fraction—compounds of lower polarity that require a higher organic-solvent proportion for efficient solubilization. This interpretation is directly supported by studies in which hydroethanolic extraction of parsley yielded the highest polyphenol and flavonoid contents and the strongest antioxidant activity among the media tested [29], as well as by optimisation work identifying ethanol as the solvent of choice for recovering apigenin from parsley leaves owing to its polarity match with the flavonoid structure [30].

For parsley ABTS, uniquely within the dataset, solvent was not the dominant factor; instead, extraction method (*p* < 0.001) and time (*p* < 0.01) governed the response, with UAE conditions frequently occupying the highest statistical group (e.g., 31.04 ± 2.90 mg/g at 80 °C/30 min in water and 33.71 ± 1.46 mg/g at 60 °C/30 min in 50% ethanol (*v*/*v*)). The prominence of the mechanical method here is consistent with the acoustic-cavitation mechanism of UAE, which enhances cell-wall disruption and mass transfer and has been reported to increase both phenolic recovery and associated antioxidant activity relative to other techniques [31]. This effect may be attributed, at least in part, to the acoustic cavitation associated with UAE, which is reported to disrupt cell walls and enhance mass transfer [11]; however, this mechanism was not directly assessed here (e.g., by microscopy or extraction-kinetics measurements) and remains a plausible interpretation rather than a demonstrated cause.

A central observation is that the extraction condition maximising TPC did not, in general, coincide with that maximising the radical-scavenging activity, and that the DPPH and ABTS assays themselves did not always rank with the conditions concordantly. In coriander, for instance, the aqueous extracts richest in total phenolics did not yield the highest ABTS values; the strongest ABTS responses were instead recorded for hydroethanolic UAE extracts (up to 87.22 ± 5.85 mg/g at 80 °C/15 min in 50% ethanol (*v*/*v*), UAE), even though these conditions were intermediate or low in TPC. This dissociation is mechanistically coherent with the differing solvent compatibility of the two radicals: DPPH is soluble predominantly in organic media and therefore preferentially detects fewer polar antioxidants, whereas ABTS•+ is compatible with both aqueous and organic media and captures hydrophilic and lipophilic antioxidants alike. Reports on hydroethanolic extraction have shown that raising the ethanol proportion can increase DPPH and ABTS scavenging severalfold, even where the phenolic profile shifts qualitatively, underlining that antioxidant potency is governed by the nature of the compounds extracted rather than by their total quantity alone.

The imperfect and sometimes negative association between total phenolic content and radical-scavenging capacity observed here is not anomalous; comparable discrepancies between the Folin–Ciocalteu response and DPPH or ABTS outcomes have been documented across a range of plant matrices and attributed to the unequal molar contribution of individual phenolics to each assay and to the differing sensitivity of the assays to specific structural classes [22]. These results reinforce the recommendation to report phenolic content and at least two mechanistically distinct antioxidant assays in parallel, since optimisation against a single response would misrepresent the functional value of the extracts.

Taken together, the results demonstrate that parsley and coriander residues, despite belonging to the same botanical family, require distinct extraction strategies. Among the conditions tested, coriander residues yielded the highest phenolic content with water at moderate temperature to maximise the recovery of their predominantly hydrophilic phenolics, whereas parsley residues require a predominantly ethanolic medium, and benefit from ultrasound assistance where antioxidant capacity is the target. The substantial difference in absolute TPC between the two matrices further indicates that coriander residues are the richer source of extractable total phenolics under the conditions tested, while parsley extracts derive a comparatively greater share of their antioxidant capacity from a smaller, more lipophilic compound pool. This species-dependence confirms that extraction protocols validated for one aromatic herb cannot be transferred uncritically to a botanically related one, and that matrix-specific optimisation is a prerequisite for the efficient valorisation of aromatic-herb processing residues within a circular-bioeconomy framework.

Beyond the main effects, the full factorial model revealed that the extraction factors did not act independently: numerous two-way interactions were statistically significant, and their pattern differed between species and assays (Table 3). In coriander, TPC was governed by strong Temperature × Solvent (F = 28.4, *p* < 0.001) and Temperature × Method (F = 16.6, *p* < 0.001) interactions, indicating that the influence of temperature depended on which solvent and method were used, consistent with the near equivalence of 60 and 80 °C in water but not in the ethanolic systems. For the antioxidant assays, the Method × Solvent interaction was dominant in coriander (DPPH F = 22.2; ABTS F = 27.0; *p* < 0.001), showing that the advantage of UAE over MAE materialized only in specific solvents.

Parsley displayed the most pronounced interactions of the dataset. Its DPPH response was shaped by a very strong Method × Solvent interaction (F = 60.2, *p* < 0.001) together with significant Temperature × Time (F = 12.3), Temperature × Solvent (F = 9.7), and Time × Solvent (F = 13.0) terms (all *p* < 0.001). The significant Temperature × Time interaction is particularly noteworthy, as it confirms quantitatively the coupled temperature–time behaviour anticipated in the Introduction—the best-performing condition among those tested was itself conditional on the temperature applied, rather than being independently fixed. Parsley ABTS was likewise dominated by a Temperature × Solvent interaction (F = 47.5, *p* < 0.001). These results collectively demonstrate that single-factor optimisation would be misleading for these matrices, and that the response surface is governed by factor combinations rather than by any factor in isolation.

In parsley, the effect of extraction time was not uniform but depended on the solvent system, as shown by the significant time × solvent interaction (DPPH, F = 13.0, *p* < 0.001). In the aqueous system, the two extraction times produced markedly different responses depending on temperature, whereas in the ethanolic systems the influence of time was smaller and more consistent. In particular, with 80% ethanol—the solvent that yielded the highest phenolic and antioxidant values for parsley—a shorter extraction time (15 min) tended to be sufficient and prolonging the extraction to 30 min brought no clear benefit and, in some cases, lower values. This may reflect a balance between compound release and the degradation of thermolabile constituents over longer exposure. Such a pattern suggests that the optimal extraction time for parsley residues cannot be defined independently of the solvent, and that shorter times combined with a more ethanolic medium are preferable when antioxidant recovery is the target. As noted, these interaction effects are exploratory and describe trends observed in the tested conditions.

To aid interpretation of the numerical values in Table 3, the most relevant interactions are shown as interaction plots in Figure 1. The non-parallel profiles confirm the exploratory patterns described above. For coriander TPC (Figure 1a), the decline in phenolic content with increasing ethanol proportion was steeper at 60 °C than at 80 °C, so that the two temperatures diverged in the ethanolic systems although nearly equivalent in water. For coriander ABTS (Figure 1b), the advantage of UAE over MAE was apparent only in the 50% ethanol system where UAE reached its highest values, whereas the two methods behaved similarly in water and in 80% ethanol. In parsley, the Method × Solvent interaction for DPPH (Figure 1c) was the most pronounced of the dataset: UAE and MAE gave comparable responses in water but diverged markedly in the ethanolic systems, with UAE increasingly favoured as the ethanol proportion rose. For parsley ABTS (Figure 1d), the two temperatures produced opposite solvent trends, the 60 °C and 80 °C profiles crossing between water and the ethanolic media. Consistent with Section 2.8, these plots are presented to illustrate the direction of the observed trends rather than as confirmatory effects.

The phenolic contents obtained in this study are consistent with, and in several cases exceed, values reported for the two species. For coriander, the best aqueous residue extracts (up to 49.7 mg GAE/g DW) were approximately twice the value of about 24 mg GAE g^−1^ reported for fresh coriander leaves [32], indicating that the processing residues are not a phenolic-depleted fraction but a matrix rich in extractable phenolics in their own right. Solvent-dependent maxima of the same order have likewise been described for coriander seeds [8], where polar solvents afforded both the highest total phenolic content and the greatest antioxidant activity, in agreement with the aqueous optimum observed here.

For parsley, the maximum residue values obtained in 80% ethanol (*v*/*v*) (up to 7.2 mg GAE/g DW) fall within the broad range of roughly 2.1–12.5 mg GAE/g compiled across several parsley leaf studies and are of the same order as the 5.1 mg GAE/g reported for parsley leaf extract [33,34]. The upper bound of that range corresponds to the aqueous parsley extract reported by Epifanio et al. [33]. The values remain, as expected, below the 42 mg GAE/g obtained by Tang et al. [35], who used dichloromethane, a non-food-grade solvent that lies outside the scope of a green-extraction approach and is therefore not directly comparable. The approximately sevenfold difference between the two species observed in the present work is thus not an artefact of the extraction protocol but reflects a genuine compositional divergence that is independently corroborated by these published values.

A direct numerical comparison should nonetheless be made with caution, as the cited studies differ in the matrix analysed (fresh leaf versus processing residue), in the extraction solvent and technique, and on the basis of which the results are expressed (per gram of dry plant material versus per gram of dried extract).

Integrating the three assays with the factorial analysis allows a practical extraction strategy to be defined for each species. Because the total phenolic content and the two radical-scavenging assays were not always maximised by the same conditions, the recommended settings are framed according to the target output, phenolic yield, or antioxidant capacity, wherever the optima diverged.

For coriander, solvent composition was the decisive factor (*p* < 0.001 for all three assays), but it acted in opposite directions depending on the response. The highest total phenolic content was obtained with water (solvent-averaged 45.5 mg GAE/g DW), whereas both DPPH and ABTS activities peaked with the 50% ethanol (*v*/*v*) hydroethanolic mixture (ABTS solvent-averaged 60.0 vs. 26.3 mg/g DW in water). This dissociation indicates that water extracts a larger total phenolic pool, while the hydroethanolic mixture recovers the fraction with greater radical-scavenging capacity, a pattern consistent with reports that intermediate ethanol–water mixtures favour antioxidant-active phenolics, since 50% ethanol (*v*/*v*) has repeatedly been found to enhance extraction efficiency relative to pure water or pure ethanol [28,36,37]. Temperature had a significant but minor effect, favouring 60 °C for phenolic content, while extraction time was never significant; the extraction method mattered only for ABTS, where UAE was superior (*p* < 0.001). Accordingly, the best-performing condition for coriander among those tested was water, 60 °C, 15 min when phenolic yield is the goal, or with the 50% ethanol (*v*/*v*) mixture at 60 °C for 30 min under UAE when antioxidant capacity is prioritized. The advantage of ultrasound for antioxidant recovery agrees with the studies attributing enhanced release of active compounds to acoustic cavitation and improved cell-wall disruption [11].

For parsley, the factors converged on a single strategy. The 80% ethanol (*v*/*v*) solvent maximised both TPC and DPPH (*p* < 0.001), consistent with the more lipophilic character of parsley phenolics such as apigenin and its derivatives [5,6]; ultrasound significantly improved DPPH and ABTS (*p* < 0.001), and the higher temperature (80 °C) significantly increased phenolic yield (*p* < 0.001), while extraction time was significant only for ABTS, favouring 30 min. A single condition, 80% ethanol (*v*/*v*), 80 °C, UAE, therefore, approaches the optimum for all three responses, with the extraction time adjusted to 15 min when phenolic yield is the priority or 30 min when antioxidant capacity is targeted. The consistent superiority of ultrasound for parsley aligns with green-extraction studies reporting that UAE maximises antioxidant recovery from herb and by-product matrices [31]. Additionally, the effectiveness of a short, hot extraction mirrors the optimisations of agro-industrial residues, where MAE/UAE optima of 15 min at around 80 °C with a mid-range ethanol fraction were reported for phenolic and antioxidant recovery from tomato-seed waste [38].

Overall, these results translate into clear, matrix-specific protocols: coriander requires a deliberate choice of solvent according to whether phenolic quantity or antioxidant quality is desired, whereas parsley accedes to a single, robust condition. In both cases, the practical recommendation departs from any assumption of a universal protocol, underscoring that extraction optimisation must be conducted per matrix even among botanically related species [15,16].

## 4. Conclusions

Parsley and coriander processing residues proved to be valuable sources of phenolic compounds with antioxidant activity, but their recovery was governed by markedly different extraction requirements despite the two species belonging to the same botanical family. Across both matrices, solvent composition was consistently the most influential factor, yet its optimum was species-specific: coriander residues released their predominantly hydrophilic phenolics most efficiently in water at moderate temperature, whereas parsley residues required a predominantly ethanolic medium, in agreement with their respective phytochemical profiles. Extraction temperature and time played comparatively minor roles, and the extraction method became decisive mainly for the antioxidant capacity of parsley, where ultrasound assistance was advantageous.

A central outcome of this work is that maximising total phenolic content did not guarantee maximal antioxidant capacity, and that the DPPH and ABTS assays captured complementary fractions of the antioxidant pool rather than duplicating one another. This reinforces the importance of evaluating phenolic yield alongside at least two mechanistically distinct antioxidant assays when characterising plant extracts. Taken together, the results demonstrate that extraction protocols cannot be transferred uncritically between related species, and that matrix-specific optimisation is a prerequisite for the efficient, sustainable valorisation of aromatic-herb residues. Future work should confirm the identity of the individual compounds responsible for the observed activity—for instance, through chromatographic profiling—and assess the stability and functionality of the optimised extracts in food or cosmetic applications. As only two Apiaceae species were evaluated, the conclusion that extraction protocols cannot be transferred directly between related species should be extrapolated to other umbelliferous plants with caution and warrants confirmation across a broader set of species.

## Figures and Tables

**Figure 1:**
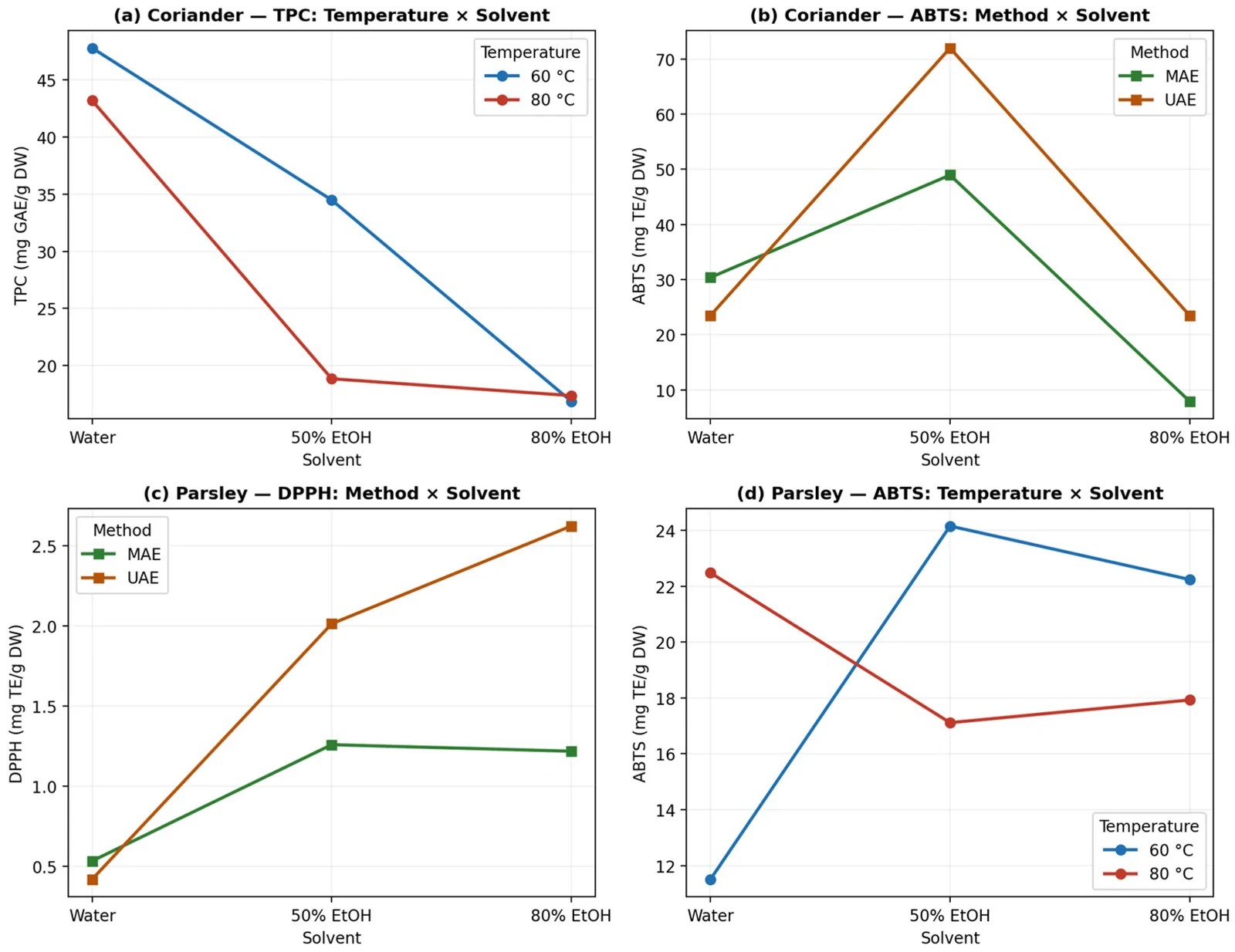
Interaction plots for the most relevant two-way interactions identified in Table 3, based on condition mean responses: (**a**) coriander TPC, Temperature × Solvent; (**b**) coriander ABTS, Method × Solvent; (**c**) parsley DPPH, Method × Solvent; (**d**) parsley ABTS, Temperature × Solvent. Non-parallel or crossing lines indicate an interaction between the two factors. Plots are presented for descriptive, exploratory interpretation (see Section 2.8).

**Table 1:** Total phenolic content (TPC), DPPH, and ABTS of coriander (*Coriandrum sativum* L.) residue extracts across the 24 extraction conditions. All responses are expressed as mean ± standard deviation, in mg gallic acid equivalents (GAE)/g DW for TPC, and mg Trolox equivalents (TE)/g DW for DPPH and ABTS.

Temperature (°C)	Time (min)	Method	Solvent	TPC (mg GAE/g DW)	DPPH (mg Trolox/g DW)	ABTS (mg Trolox/g DW)
60	15	MAE	water	49.71 ± 0.73	34.75 ± 0.59	31.17 ± 1.78
60	30	MAE		49.65 ± 0.92	26.27 ± 0.79	43.06 ± 1.77
80	15	MAE		39.16 ± 0.29	28.92 ± 0.63	17.80 ± 0.47
80	30	MAE		49.32 ± 1.21	27.42 ± 0.20	29.46 ± 0.42
60	15	UAE		43.03 ± 1.40	26.71 ± 0.60	24.51 ± 1.94
60	30	UAE		48.64 ± 1.39	28.60 ± 0.21	28.51 ± 1.04
80	15	UAE		40.86 ± 1.47	28.63 ± 0.67	26.26 ± 1.54
80	30	UAE		43.35 ± 0.65	19.43 ± 0.91	10.24 ± 1.00
60	15	MAE	50% ethanol (*v*/*v*)	40.42 ± 1.34	33.22 ± 0.56	41.88 ± 4.17
60	30	MAE		35.35 ± 0.24	28.81 ± 2.35	40.79 ± 0.77
80	15	MAE		19.55 ± 0.40	31.50 ± 0.60	43.19 ± 2.71
80	30	MAE		10.14 ± 0.91	30.09 ± 0.53	69.90 ± 5.21
60	15	UAE		24.38 ± 1.03	38.61 ± 3.63	54.36 ± 3.95
60	30	UAE		38.04 ± 1.64	41.59 ± 3.76	75.44 ± 6.53
80	15	UAE		25.05 ± 0.72	33.73 ± 0.31	87.22 ± 5.85
80	30	UAE		21.58 ± 0.15	43.15 ± 1.03	72.03 ± 3.48
60	15	MAE	80% ethanol (*v*/*v*)	23.40 ± 0.24	16.24 ± 0.71	6.71 ± 0.65
60	30	MAE		21.34 ± 1.36	30.60 ± 1.28	6.94 ± 0.56
80	15	MAE		19.10 ± 1.09	14.81 ± 1.15	9.50 ± 1.88
80	30	MAE		22.55 ± 0.75	18.45 ± 0.48	8.74 ± 0.71
60	15	UAE		13.26 ± 0.28	21.67 ± 1.90	13.13 ± 0.51
60	30	UAE		10.52 ± 0.68	17.69 ± 1.11	32.92 ± 1.03
80	15	UAE		12.91 ± 0.70	11.27 ± 0.97	22.44 ± 0.97
80	30	UAE		15.94 ± 1.07	13.48 ± 1.24	25.14 ± 1.95

**Table 2:** Total phenolic content (TPC), DPPH, and ABTS of parsley (*Petroselinum crispum* (mill.) Fuss) residue extracts across the 24 extraction conditions. All responses are expressed as mean ± standard deviation, in mg gallic acid equivalents (GAE)/g DW for TPC, and mg Trolox equivalents (TE)/g DW for DPPH and ABTS.

Temperature (°C)	Time (min)	Method	Solvent	TPC (mg GAE/g DW)	DPPH (mg Trolox/g DW)	ABTS (mg Trolox/g DW)
60	15	MAE	water	2.21 ± 0.04	0.67 ± 0.02	13.35 ± 0.68
60	30	MAE		1.23 ± 0.10	0.57 ± 0.01	13.41 ± 0.39
80	15	MAE		0.82 ± 0.02	0.45 ± 0.06	24.58 ± 1.82
80	30	MAE		3.19 ± 0.10	0.50 ± 0.03	18.75 ± 0.31
60	15	UAE		2.65 ± 0.09	0.26 ± 0.02	10.65 ± 0.74
60	30	UAE		0.86 ± 0.02	0.44 ± 0.02	8.14 ± 0.61
80	15	UAE		0.97 ± 0.02	0.54 ± 0.02	16.34 ± 1.38
80	30	UAE		4.93 ± 0.21	0.50 ± 0.00	31.04 ± 2.90
60	15	MAE	50% ethanol (*v*/*v*)	2.36 ± 0.04	0.94 ± 0.08	11.54 ± 0.68
60	30	MAE		1.64 ± 0.17	1.10 ± 0.05	24.01 ± 0.76
80	15	MAE		3.98 ± 0.07	1.10 ± 0.06	11.53 ± 0.86
80	30	MAE		3.78 ± 0.23	1.74 ± 0.10	21.60 ± 0.90
60	15	UAE		2.13 ± 0.06	0.82 ± 0.05	27.35 ± 2.22
60	30	UAE		1.99 ± 0.07	2.16 ± 0.17	33.71 ± 1.46
80	15	UAE		3.80 ± 0.11	2.22 ± 0.07	19.38 ± 1.35
80	30	UAE		4.04 ± 0.20	2.17 ± 0.15	22.59 ± 0.55
60	15	MAE	80% ethanol (*v*/*v*)	3.69 ± 0.22	1.15 ± 0.02	18.57 ± 0.70
60	30	MAE		6.57 ± 0.13	1.18 ± 0.02	17.26 ± 1.47
80	15	MAE		7.18 ± 0.29	1.19 ± 0.04	17.89 ± 0.32
80	30	MAE		5.27 ± 0.08	1.36 ± 0.06	13.49 ± 0.74
60	15	UAE		5.97 ± 0.15	2.59 ± 0.15	27.17 ± 2.16
60	30	UAE		5.62 ± 0.03	2.75 ± 0.23	27.18 ± 2.34
80	15	UAE		7.15 ± 0.12	2.84 ± 0.19	20.26 ± 1.14
80	30	UAE		4.28 ± 0.12	2.04 ± 0.08	21.46 ± 1.13

**Table 3:** Exploratory factorial ANOVA of the effects of extraction temperature (Temp.), time, method, and solvent, and their interactions, on the total phenolic content (TPC), DPPH and ABTS radical-scavenging activities of parsley and coriander residue extracts. Values are F-statistics; significance is indicated as: *** *p* < 0.001; ** *p* < 0.01; * *p* < 0.05; ns = not significant (*p* ≥ 0.05).

	Temp.	Time	Method	Solvent	Temp. × Time	Temp. × Method	Temp. × Solvent	Time × Method	Time × Solvent	Method × Solvent
Coriander										
TPC	49.0 (***)	2.9 (ns)	16.2 (***)	364.4 (***)	0.2 (ns)	16.6 (***)	28.4 (***)	4.8 (*)	3.7 (*)	10.3 (***)
DPPH	21.2 (***)	0.7 (ns)	0.0 (ns)	151.4 (***)	0.0 (ns)	0.2 (ns)	4.7 (*)	0.0 (ns)	9.0 (***)	22.2 (***)
ABTS	1.5 (ns)	9.0 (**)	31.9 (***)	229.4 (***)	3.1 (ns)	0.6 (ns)	19.1 (***)	3.9 (ns)	0.4 (ns)	27.0 (***)
Parsley										
TPC	17.8 (***)	0.3 (ns)	0.7 (ns)	83.7 (***)	0.2 (ns)	0.0 (ns)	3.4 (*)	1.4 (ns)	2.5 (ns)	0.2 (ns)
DPPH	7.5 (**)	6.4 (*)	198.2 (***)	269.0 (***)	12.3 (***)	0.0 (ns)	9.7 (***)	0.6 (ns)	13.0 (***)	60.2 (***)
ABTS	0.0 (ns)	19.3 (***)	52.5 (***)	6.9 (**)	0.6 (ns)	3.3 (ns)	47.5 (***)	2.8 (ns)	15.0 (***)	17.7 (***)

## Data Availability

The original contributions presented in this study are included in the article. Further inquiries can be directed to the corresponding author.
